# Longitudinal Imaging of the Premalignant Tumor Microenvironment Reveals Transient Myeloid States Predictive of Tumor Fate

**DOI:** 10.1101/2025.11.02.686127

**Published:** 2025-11-04

**Authors:** Thomas D. Madsen, Desu Chen, Maria Hernandez, Sarah Hammoudeh, Marco Heydecker, Emily Chen, Salma Abu-Elnaj, Noemi Kedei, Weiye Wang, Roberto Weigert

**Affiliations:** 1 Laboratory of Cellular and Molecular Biology, Center for Cancer Research, National Cancer Institute, National Institutes of Health, Bethesda, MD, USA; 2 Copenhagen Center for Glycomics, University of Copenhagen, Department for Cellular and Molecular Medicine, Copenhagen, Denmark; 3 Spatial Imaging Technology Resource, Center for Cancer Research, National Cancer Institute, National Institutes of Health, Bethesda, MD, USA

**Keywords:** Premalignant lesions, Spontaneous regression, Intravital microscopy, Myeloid Niche, Cxcl9, Cxcl10, Tumor metabolism, M2 macrophage, Tumor-Associated Macrophage, Immune checkpoint inhibitor, Tumor microenvironment

## Abstract

The spontaneous regression of cancer lesions demonstrates the potential of immune surveillance; yet, these transient events have remained inaccessible to systematic study. Using longitudinal intravital microscopy in a carcinogen-induced model of head and neck cancer, we tracked premalignant lesions within the same animals for 24 weeks at single-cell resolution. This approach revealed three trajectories: progression, stability, or regression, and enabled dissection of the immune dynamics underlying each fate. Lesion outcome was dictated by the spatial organization of myeloid-derived antigen-presenting cells: regressing lesions were characterized by dense clusters of myeloid-derived cells associated with CXCL9^+^/CXCL10^+^ expression and T cell recruitment, whereas progressing lesions displayed sparse, non-clustered infiltration. Remarkably, transient myeloid clusters arose prior to any detectable lesion formation and consistently marked regions that would later develop into premalignant lesions. These findings identify spatiotemporal myeloid organization as an early determinant of tumor fate and provide a mechanistic framework for predicting and intercepting cancer at its inception.

## Summary Sentence

Early myeloid architecture dictates cancer fate: dense CXCL9^+^/CXCL10^+^ clusters with T-cell enrichment accompany regression, whereas sparse infiltration predicts progression. Transient pre-lesional myeloid clusters emerge at future tumor sites, revealing immune organization as an early determinant of malignancy.

## Introduction

Cancer arises from a complex interplay of environmental exposures and somatic mutations, and remains a major global health burden, with incidence and mortality continuing to rise worldwide (Bray et al. 2024). Although spontaneous regression of malignant lesions has been documented clinically, these events are transient and therefore rarely accessible for systematic study (Lau and Ferozepurwalla 2024; Jessy 2011; Radha and Lopus 2021). The inability to capture and follow regression in real time has limited mechanistic insight, despite clear evidence that the body possesses an intrinsic capacity to eliminate cancer without therapeutic intervention. Understanding these dynamics, particularly in the earliest stages of tumorigenesis, could inform preventive and therapeutic strategies that harness endogenous immune surveillance. Immune checkpoint blockade (ICB) illustrates the potential of restoring immune control by blocking T-cell inhibitory receptors such as PD1/PD-L1 and CTLA4 (Bagchi et al. 2021). Yet only a minority of patients respond to ICB (Haslam and Prasad 2019; Haslam et al. 2020), and adverse immune-related effects remain a major limitation (Johnson et al. 2022). While the absence of T-cell infiltration is a hallmark of non-responsive “cold” tumors, even “hot” tumors with abundant T-cell infiltration show variable outcomes, suggesting that additional immune and stromal components shape therapy response (Galon and Bruni 2019; Sahu et al. 2022). Recent studies have highlighted the role of the innate immune system, showing that specialized myeloid–lymphoid niches, defined by the spatial arrangement of antigen-presenting cells and T cells, predict favorable outcomes across several cancer types (Pelka et al. 2021; Magen et al. 2023; Duraiswamy et al. 2021; Oh et al. 2025). These findings underscore the importance of considering not only the identity of infiltrating cells but also their spatial and temporal dynamics within the tumor microenvironment.

Longitudinal intravital microscopy provides a unique opportunity to study these dynamics by repeatedly imaging living tissues at single-cell resolution. Previous work has applied this approach to characterize invasion (Hammoudeh et al. 2024; Harper et al. 2016; Harney et al. 2015; Chepizhko et al. 2025), metastasis (Borriello et al. 2022; Sharma et al. 2021), tumor cell metabolism (Hemalatha et al. 2025), and immune responses in established tumors (Ng et al. 2024; Harney et al. 2015; Arlauckas et al. 2017; Ritsma et al. 2013; Mempel et al. 2006; Nakasone et al. 2012). However, most of these efforts have relied on xenografts or genetically engineered mouse models that capture late-stage diseases with known lesion outcome, leaving the immune dynamics of premalignant lesions and the cellular mechanisms that determine initial lesion growth largely unexplored. To address this gap, we adapted intravital microscopy to follow the premalignant and neoplastic microenvironment in the tongues of 4-nitroquinoline-1-oxide (4NQO), a water-soluble carcinogen that induces stepwise transformation from dysplasia to squamous cell carcinoma (Vitale-Cross et al. 2012; 2009; Amornphimoltham et al. 2017), This model of head and neck squamous cell carcinoma (HNSCC) closely mirrors the mutational spectrum of human disease and shows partial responsiveness to PD-1 blockade (Sequeira et al. 2020; Wang et al. 2017). Using this carcinogen approach, we recently discovered that within the same animal, a subset of 4NQO-induced lesions spontaneously regresses while others progress to carcinoma (Wang et al., man in prep). This observation reveals that local, rather than systemic, factors determine lesion fate and provides an experimental system to dissect the underlying mechanisms.

Here, we use this experimental system to define how myeloid cells behave during the earliest phases of neoplasia and how their organization influences lesion outcome. By combining longitudinal intravital microscopy with multiplex immunostaining and spatial transcriptomics, we mapped both dynamics, architecture, and molecular states of myeloid infiltrates over 24 weeks in the same animal. We find that antigen-presenting cells, myeloid-derived dendritic cells (mDCs) in particular, dominate the myeloid compartment and that their spatial organization predicts lesion fate. Clustering versus scattered distribution of these cells upon infiltration within the premalignant lesion distinguishes regressing from progressing growth trajectories. Dense clusters of mDCs, forming compact aggregates within the tumor epithelium, characterize regressing lesions and create CXCL9^+^/CXCL10^+^ chemokine-rich niches and local T cell enrichment. Smaller, scattered clusters represent an intermediate state, whereas sparse and isolated mDCs fail to support effective immune responses and correlate with lesion progression. Progressing lesions contain scattered mDCs expressing inflammatory and lipid-metabolic mediators such as MMP12 and ApoE, revealing the earliest dysregulated myeloid programs at cancer onset. Unexpectedly, transient clusters of CXCL9^+^/PD-L1^+^ mDCs emerged at discrete epithelial sites weeks before any morphological signs of lesions and consistently marked regions that later developed into premalignant lesions, revealing a spatially confined, early indicator of tumor initiation. Together, these findings demonstrate that the spatiotemporal organization of myeloid cells dictates whether premalignant lesions regress or progress and establishes a mechanistic framework for predicting tumor fate at its inception.

## Results

### Longitudinal intravital imaging reveals three trajectories of early lesion fate

**Figure 1 -:**
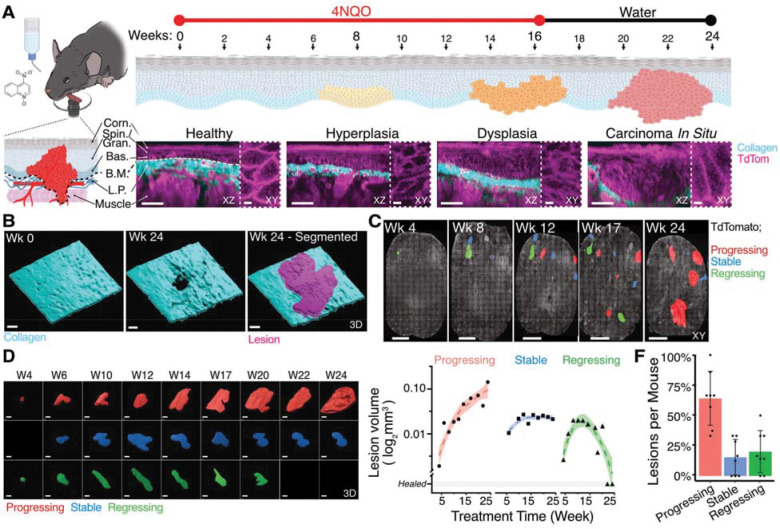
Longitudinal intravital microscopy reveals distinct trajectories of premalignant lesion fate. Eight *LysM*-Cre; *mT/mG* mice expressing membrane-bound TdTomato (all cells) and GFP (myeloid cells) were treated with 50 μg/mL 4NQO in drinking water for 16 weeks, followed by an 8-week recovery period. The rostral two-thirds of the ventral tongue were imaged biweekly by intravital two-photon microscopy. **(A)** Schematic of the imaging workflow and tongue epithelial layers. Fluorescence images show high-resolution optical sections of lesions at different stages of premalignant progression. TdTomato gamma is set to 1.5 to outline epithelial morphology. Scale bar, 50 μm. Inserts show the morphology of epithelial cells along the basedment membrane. Scale bar, 5 μm **(B).** Three-dimensional segmentation of the lamina propria (collagen) and lesion epithelium, highlighting collagen displacement and altered cell morphology. Scale bar, 100 μm. **(C)** Whole-tongue image showing segmented lesions distributed across distinct regions of the ventral surface. Colored outlines indicate lesions with different growth behaviors. Scale bar, 1 mm. **(D)** Temporal 3D segmentation of three representative lesions illustrating the three trajectories—progression, regression, and stability. Loess fit of log_2_-transformed lesion volumes over time shows exponential growth for progressing lesions, while stable and regressing lesions show no growth trend. Scale bar, 200 μm. **(E)** Summary of lesion outcomes across all mice. Fifty-two lesions (~500 timepoints) were recorded: 28 progressed, 8 stabilized, and 11 regressed (6 partial, 5 complete). On average, each mouse developed ~4 progressing, 1.5 regressing, and 1 stable lesion. One regressing and four progressing lesions that evolved into benign papillomas were excluded from analysis. 4-NQO, 4-nitroquinoline-1-oxide; Corn, corneal layer; Spin, spinous layer; Gran, granular layer; Bas, basal layer; BM, basement membrane; LP, lamina propria.

To investigate the interaction between myeloid cells and premalignant lesions, we employed the carcinogen-induced 4-nitroquinoline-1-oxide (4NQO) tongue model of HNSCC (Fig. 1A). This model offers several advantages: (1) It can be performed in immunocompetent mice allowing study of the inflammatory and immune context of carcinogenesis; (2) lesions arise through random mutagenesis and recapitulate the mutational spectrum of early human disease (Sequeira et al. 2020); (3) the relatively slow onset of tumors (8–16 weeks) enables detailed analysis of early pathological stages (Sagheer et al. 2021; Vitale-Cross et al. 2012); and (4) the tongue is the principal site for lesion formation (Tang et al. 2004; Czerninski et al. 2009), which is readily accessible for non-invasive and repeated imaging (Amornphimoltham et al. 2017) (Wang et al., man. in prep).

For longitudinal intravital microscopy (IVM), we used LysM-cre; mT/mG dual reporter mice in which myeloid cells express membrane-bound GFP and all other cells membrane-bound TdTomato. This configuration allowed simultaneous visualization of myeloid cells (GFP^+^), the epithelium (TdTomato^+^), and the collagen-rich interstitial matrix through second harmonic generation (SHG) (Brown et al. 2003). Because the exact site of tumor initiation cannot be predicted in carcinogen models, we imaged two-thirds of the ventral tongue biweekly to a depth of 150 μm, capturing tissues before, during, and after carcinogenesis.

Following the 4NQO treatment, premalignant lesions were identified based on altered morphology and collagen deformation (Fig. 1B–D). Three-dimensional segmentation confirmed distinct lesion boundaries and enabled quantification of lesion growth kinetics (Fig. S1A–D, See Method Section). Lesions segregated into three categories: progressing (64 ± 22%), regressing (20 ± 17%; partial or complete), and stable (16 ± 15%) (Fig. 1E). Progressing lesions exhibited an average volumetric growth rate of 24 ± 10% per week (Fig. S1E). Each animal developed on average 3–5 progressing lesions, 1–2 regressing lesions, and one stable lesion. The coexistence of all three trajectories within the same animal ruled out systemic factors and framed a platform to dissect the local immune determinants of lesion fate. These proportions are consistent with observations from the companion study (Wang et al., man. in prep)., which reported comparable distributions across different mouse strains, underscoring the robustness of this phenomenon.

Together, these results establish a platform for dissecting the cellular and molecular mechanisms governing lesion fate in vivo.

### Infiltrating Myeloid Behavior and Tumor Metabolism Predict Lesion Fate

**Figure 2 -:**
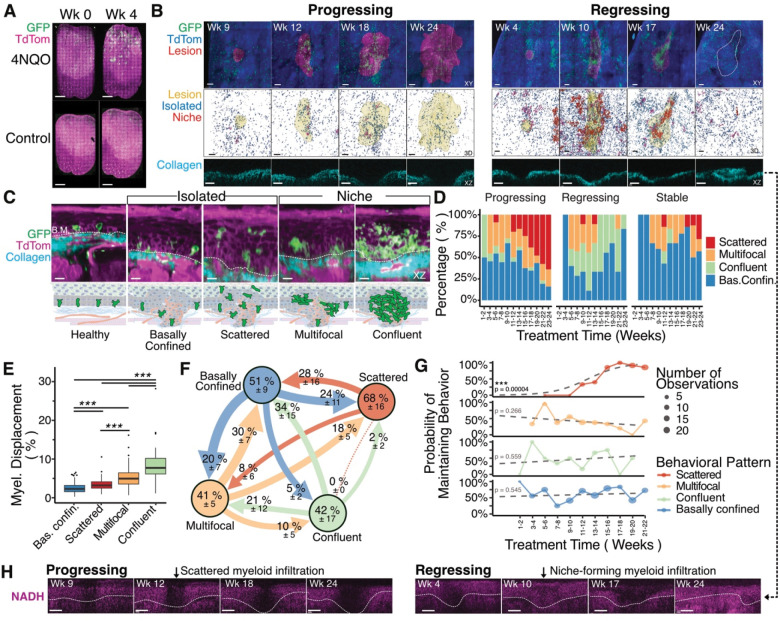
Transient myeloid behavioral states predict the progression or regression of premalignant lesions. Myeloid cells (GFP+) were imaged in the tongues of *LysM*-Cre; *mT/mG* mice during the 24-week 4NQO treatment regimen described in Fig. 1. **(A)** Whole-tongue scans before and after 4 weeks of 4NQO treatment show increased myeloid infiltration following carcinogen exposure. Control mice treated with vehicle alone displayed a stable baseline of myeloid cells at both time points (see Fig. S2 for the complete time course). Scale bar, 1 mm. **(B)** Sum projections of lesion epithelium showing myeloid infiltration in a progressing lesion (left) and a regressing lesion (right). Middle panels: 3D segmentation in the GFP channel of isolated (blue; < 5000 μm^3^) and niche-forming (red; > 5000 μm^3^) myeloid cells reveals larger and more frequent niches in regressing lesions. Bottom: cross-sectional view of the collagen layer showing collagen restoration after lesion clearance. Scale bar, 100 μm. **(C)** Cross-sectional views illustrating the four principal intratumoral myeloid behaviors compared with healthy tissue. Scale bar, 100 μm. **(D)** Bar plots showing the predominant myeloid organization pattern observed within lesions over time, stratified by lesion outcome. **(E)** Quantification of myeloid displacement (volume of infiltrating myeloid cells / total lesion volume, %) stratified by myeloid behavior. Statistical comparisons were performed using the Kruskal–Wallis test with pairwise Wilcoxon post hoc testing. **(F)** Transition map showing the probability of changes in myeloid behavior between consecutive imaging sessions (2 weeks apart). Numbers represent the probability of behavior change (arrows) or maintenance (inside circles) expressed as mean ± SEM (n = 6–67 transitions per mouse, 8 mice total). **(G)** Line graph depicting changes in the probability of maintaining each myeloid behavior over time. Associations between behavioral maintenance and lesion age were tested using the Cochran–Armitage trend test. **(H)** Cross-sectional NADH images from the same lesions as in (A), showing a decline in NADH intensity preceding myeloid infiltration in progressing lesions and occurring during infiltration in regressing lesions. Representative images are shown from three progressing and three regressing lesions. Scale bar, 100 μm.

In the healthy tongue, myeloid cells were sparsely distributed across distinct tissue compartments. A few dendritic cell (DC)–like cells with branched protrusions were present in the epithelial layer, macrophage-like cells occupied the lamina propria, and neutrophil-like cells were confined to blood vessels. Immunofluorescence confirmed these identities: GFP^+^ cells within the epithelium corresponded to Langerhans cells (GFP^+^/Langerin^+^), interstitial cells expressed CD68 consistent with macrophages, and occasional Ly6G^+^ neutrophils were restricted to the vasculature (Fig. S2A–C). A subset of perivascular GFP^+^ cells lacking CD45, CD68, MHCII, and Langerin expression remained immobile throughout 24 weeks of imaging (Fig. S2D), consistent with neuronal identity and previous reports of *LysM*-Cre activity in neurons (Orthgiess et al. 2016). Upon 4NQO treatment, a pronounced influx of myeloid cells was observed throughout the tongue (Fig. 2A, S2E). This response was specific to carcinogen exposure, as control mice imaged under identical conditions showed neither lesion formation nor myeloid recruitment, confirming that the infiltration was not induced by the imaging procedure.

We next focused on myeloid cells infiltrating the lesion epithelium and examined how their spatial organization correlated with lesion trajectory—progression, regression, or stability. Qualitative analysis revealed distinct behavioral patterns: infiltrating myeloid cells in progressing lesions appeared largely isolated, whereas those in regressing lesions assembled into multicellular clusters or niches (Fig. 2B). To capture these arrangements semi-quantitatively, we classified myeloid organization into four morphological states reflecting increasing interaction and density within the lesion (Fig. 2C). In the basally confined state, cells aligned along the basement membrane, similar to those in healthy tissue. The scattered state consisted of individual cells that migrated into the suprabasal epithelium, losing contact with the basement membrane. The multifocal state contained several small clusters of myeloid cells, while the confluent state was defined by large, continuous aggregates forming a single immune niche that occupied a substantial portion of the lesion.

At early time points, lesions across all trajectories primarily exhibited basally confined myeloid cells or transient multifocal niches (Fig. 2D, S1F), indicating that initial epithelial transformation alone is insufficient to trigger a robust immune response. Over time, distinct temporal patterns emerged that correlated with lesion fate. Progressing lesions developed a scattered organization, with cells displaying dendritic morphology and active antigen-scavenging behavior. In contrast, regressing lesions formed confluent niches composed of cells containing endocytosed TdTomato signal and a foamy cytoplasm, consistent with increased phagocytic activity (Fig. S1G). Stable lesions retained predominantly basally confined or multifocal myeloid arrangements, similar to early-stage organization. Quantitative analysis of myeloid infiltration, measured as the percentage of total lesion volume, revealed a monotonic increase in cell density from basally confined to scattered, multifocal, and confluent patterns (Fig. 2E, S1H–I).

Together, these findings demonstrate that 4NQO treatment induces a targeted recruitment of myeloid cells to the tongue, where their local organization evolves dynamically over the course of disease. The spatial continuum—from dense, immune-active niches driving regression to scattered, immune-ineffective infiltrates promoting progression—defines a structural framework linking immune architecture to lesion fate.

Next, we addressed a central question in tumor immunology: how dynamic are myeloid behavioral patterns within the same lesion over time? Longitudinal intravital imaging enabled noninvasive tracking of these transitions in individual lesions between consecutive imaging sessions spaced two weeks apart (Fig. 2F). Analysis of transitions between myeloid organizational states across all lesions revealed varying degrees of temporal stability. The scattered pattern was the most stable, with only 32 ± 16% of lesion time points showing a change at the next imaging session. In contrast, the multifocal and confluent niche-forming patterns were the most dynamic, altering behavior in 58 ± 17% and 59 ± 5% of time points, respectively, whereas the basally confined state showed intermediate stability, changing in 49 ± 9% of time points.

When examining the directionality of these transitions, we found that scattered, multifocal, and confluent patterns were more likely to revert to the basally confined configuration than to interconvert among infiltrative states. This suggests that the multifocal pattern represents incomplete recruitment—or a failed attempt at lesion clearance—rather than a transient phase leading to confluent, tumor-clearing organization. To further quantify these dynamics, we calculated the probability of maintaining each organizational state as a function of time (Fig. 2G). The likelihood of preserving the scattered pattern increased steadily with treatment duration, indicating that when lesions adopt this configuration at later timepoints, they reach a point of no return predictive of continued progression. In contrast, the multifocal niche-forming pattern became progressively less stable over time, which likely reflecting a developing immunosuppressive microenvironment. The basally confined and confluent configurations remained relatively constant throughout lesion evolution.

Together, these analyses reveal that myeloid spatial organization is not static but evolves dynamically within individual lesions. Scattered infiltrates that persist over multiple weeks mark irreversible progression, whereas transient confluent niches define a window of effective immune clearance during early tumor development.

Because tumor metabolism profoundly shapes immune behavior (Bill et al. 2023; Ng et al. 2024), we next examined the endogenous fluorescence of NADH as a real-time, label-free indicator of cellular metabolic activity (Porat-Shliom et al. 2014)(Wang et al man in prep). This analysis revealed a trajectory-dependent metabolic signature: progressing lesions showed an early decline in NADH intensity that preceded the onset of scattered myeloid infiltration, whereas regressing lesions maintained stable NADH levels until the appearance of confluent myeloid niches (Fig. 2H). Importantly, the regions of low NADH signal in regressing lesions coincided spatially with confluent myeloid clusters and reflected reduced NADH levels within the infiltrating cells, rather than loss of signal from tumor cells. Stable lesions maintained consistent NADH intensity throughout their lifetime (data not shown). Although the metabolic reduction observed in progressing lesions may result from, rather than cause, altered immune activity, it nevertheless provides a functional readout tightly correlated with lesion behavior. Thus, NADH dynamics serve as a real-time marker for predicting lesion trajectory and for monitoring the evolving interplay between metabolism and immune response.

### Myeloid-derived dendritic cells are the primary infiltrating myeloid cell type

**Figure 3 -:**
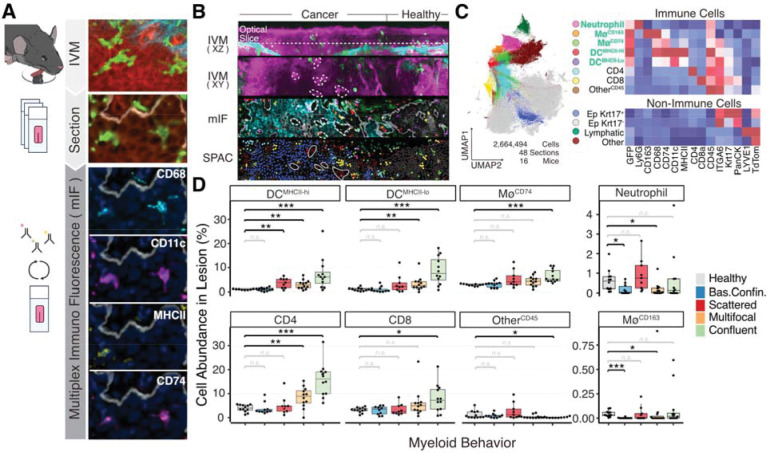
Correlative intravital microscopy and multiplex immunostaining identify tumor-infiltrating myeloid cells as antigen-presenting cells (APCs). Tongues from *LysM*-Cre; *mT/mG* mice at weeks 0 (control), 8, 16, and 24 of the 4NQO treatment regimen were imaged intravitally and immediately processed for correlative histological sectioning and multiplex immunofluorescence (mIF). **(A)** Schematic of the correlative workflow from IVM to histological sections for mIF analysis. Insets show the same two myeloid cells visualized by IVM and mIF. **(B)** Overview image of a lesion imaged by IVM (top), the corresponding mIF-stained section (middle), and projected cell identities determined by Phenograph clustering (bottom). **(C)** Unsupervised Phenograph clustering displayed as a UMAP identifying 12 major cell phenotypes. The accompanying heatmap shows the mean marker expression profile for each phenotype. **(D)** Boxplots showing the abundance of immune cell subsets quantified by mIF, stratified by intratumoral myeloid organization as determined by IVM. Pairwise Wilcoxon tests were performed to compare each myeloid organization pattern with healthy basal tissue.

To identify the cell types underlying the functional states of confluent and scattered infiltrates associated with regression and progression, respectively, we developed a correlative workflow that combined longitudinal intravital microscopy (IVM) with endpoint cyclic multiplex immunofluorescence (mIF) based on the *Iterative Bleaching Extends Multiplexity* (IBEX) protocol (Radtke et al. 2022). Tongues were cryo-embedded flat against a glass surface to preserve the ventral epithelium as an intact sheet, allowing spatial coordinates from histological sections to be directly aligned with corresponding three-dimensional intravital volumes and enabling cell-to-cell correlation between IVM and mIF datasets (Fig. 3A–B). This approach was applied to mice at weeks 8, 16 (end of carcinogen exposure), and 24, as well as untreated controls, thereby capturing lesions across multiple developmental stages.

Across these cohorts, lesion size increased, while both myeloid infiltration and intratumoral NADH intensity decreased (Fig. S3A–C), consistent with longitudinal imaging data from Fig. 2. We correlated IVM and mIF results from 50 lesions collected across these time points (Fig. S3D). Multiplex mIF images were subjected to single-cell segmentation and analyzed using the SPAC computational platform (Liu et al. 2025) (Fig. 3B–C). This approach identified 12 major cell populations. Within the LysMcre^+^/GFP^+^ myeloid compartment, we distinguished neutrophils (Ly6G^+^), CD68^+^/CD74^+^ macrophages (Mø^CD74+^), CD68^+^/CD163^+^ macrophages (Mø^CD163+^), as well as mature dendritic cells (DC^MHCII-hi^; CD11c^+^/MHCII^hi^) and immature dendritic cells (DC^MHCII-lo^; CD11c^+^/MHCII^lo^). Both dendritic cell subsets were primarily of myeloid origin (myeloid-derived DCs; mDC), as indicated by strong LysM-Cre–driven GFP expression and high CD68 levels. Additional immune populations included CD4^+^ and CD8^+^ T cells, along with a residual CD45^+^ population negative for lineage markers (designated “Other CD45^+^”), collectively representing the immune landscape of 4NQO-treated tongues.

The panel also resolved non-immune compartments, including healthy epithelium (ITGA6^+^/PanCK^+^/Krt17^−^), transformed epithelium (Krt17^+^), and lymphatic vessels (LYVE1^+^). Notably, B cells (B220^+^) were rare or absent at all time points (data not shown), consistent with previous findings in the 4NQO tongue model (Saba et al. 2022) and human HNSCC (Oh et al. 2025). Consequently, they were excluded from the final staining panel.

Across treatment time, overall immune infiltration peaked at week 8 and declined by weeks 16 and 24 (Fig. S3E–F). CD163^+^ macrophages decreased steadily over the course of treatment, consistent with findings in human HNSCC showing limited prognostic relevance of this population (Bill et al. 2023). In contrast, the “Other CD45^+^” population was markedly reduced during 4NQO exposure (weeks 8–16) but recovered by week 24, suggesting a transient sensitivity to carcinogen-induced stress. These CD45^+^ cells, localized primarily to the basal epithelium, lacked CD4, CD8, CD68, Ly6G, CD11c, and GFP expression, which likely represent resident innate lymphoid or natural killer cell populations as has been previously described in the murine tongue (Lyras et al. 2022; Saba et al. 2022; Sahu et al. 2023). Collectively, this correlative workflow enabled direct alignment of intravital and multiplex histological data, providing high-resolution identification of the myeloid and lymphoid compartments infiltrating premalignant lesions.

To define the immune composition associated with dense versus sparse myeloid infiltrates, we quantified immune cell subsets in 50 lesions from the multiplex mIF cohort and correlated them with the myeloid organizational patterns identified by IVM (Fig. 3D). Among GFP^+^ myeloid cells, mature and immature DCs together with CD74^+^ macrophages were the predominant infiltrating populations in lesions exhibiting confluent, multifocal, or scattered myeloid organization. In contrast, CD163^+^ macrophages and neutrophils were largely confined to the stroma and rarely detected within either healthy or neoplastic epithelium, showing minor but significant exclusion from lesions with basally confined or multifocal behavior. Analysis of the lymphoid compartment revealed that CD4^+^ T cells were present at similar levels in healthy tissue and in lesions with scattered myeloid organization but were markedly enriched in lesions with multifocal or confluent myeloid clustering, consistent with enhanced recruitment within chemokine-rich DC niches. CD8^+^ T cells were specifically enriched in densely clustered lesions, indicative of a cytotoxic, lesion-clearing immune response.

Unsupervised clustering of 62 lesions analyzed by mIF (50 also characterized by IVM, 12 by mIF alone) defined six immune signatures (Fig. S3G) that closely matched intravital behaviors (Fig. S3H). Signature 1, similar to healthy tissue except for slight enrichment of neutrophils and CD163^+^ macrophages, comprised 70% of lesions with basally confined myeloid organization. Signatures 4–5, enriched for CD74^+^ macrophages, mature and immature DCs, and CD4^+^ T cells, represented “hot” confluent lesions (66%), whereas Signature 3, enriched in CD74^+^ macrophages and mature DCs but lacking T cells, corresponded to “cold” scattered lesions (56%). Signature 2 showed intermediate DC and CD4^+^ T-cell levels, matching multifocal lesions (58%), and Signature 6, enriched in CD8^+^ T cells (two lesions), was considered an outlier. Despite strong concordance, some overlap reflected the transient nature of myeloid organization: 17% of dense lesions exhibited Signature 2, and 33% of scattered lesions exhibited Signature 1. Temporally, CD4^+^ T-cell–rich Signatures 4–5 predominated at weeks 8–16 but disappeared by week 24 (regressing lesions), whereas “cold” Signature 3 increased over time (progressing lesions). Signature 1 remained stable, and Signature 2 peaked at week 16 before declining (Fig. S3I).

Interestingly, neutrophils were not enriched above healthy-adjacent levels within any lesion group by mIF, despite being among the most abundant immune populations overall (Fig. S3E). These cells were largely excluded from the epithelium. To further define their role, we examined tongues after varying durations of 4NQO treatment and observed a pronounced accumulation of neutrophils in the lamina propria within the caudal part of the tongue, particularly around the deep lingual veins at early time points. Over time, the neutrophil gradually spread out rostrally while remaining confined to the lamina propria (Fig. S4A). Although neutrophils rarely infiltrated lesions, they were consistently distributed in the surrounding stroma across all treatment stages. Notably, they preferentially accumulated around lesions with reduced NADH fluorescence, making them the only immune population specifically recruited to the tumor stroma in response to this metabolic state (Fig. S4C–D). Together, these findings establish antigen-presenting cells—specifically mDCs and CD74^+^ macrophages—as the dominant infiltrating myeloid populations in 4NQO-induced lesions. Lesions with clustered DCs accompanied by T cells correspond to “hot” tumors that spontaneously regress, whereas T cell–poor lesions containing sparsely distributed antigen-presenting cells correspond to “cold” tumors that progress. Collectively, these results identify mDCs and CD74^+^-macrophages as the key immune population bridging innate and adaptive responses and demonstrate that their spatial organization and metabolic context dictate whether premalignant lesions regress or progress.

### Distinct transcriptomic programs underlie infiltrating myeloid organization

**Figure 4 -:**
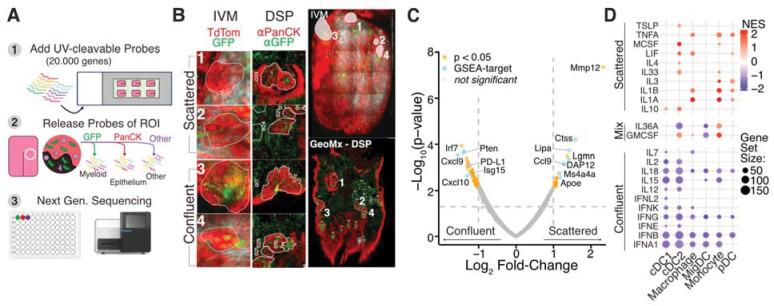
Correlative spatial transcriptomics reveals pro- and anti-tumorigenic transcriptional programs associated with scattered and clustered myeloid behaviors, respectively. The tongue of a mouse at the end of the 24-week 4NQO treatment regimen was imaged intravitally and sectioned as described in Fig. 3, followed by spatial transcriptomic analysis using the GeoMx Digital Spatial Profiling (DSP) platform. **(A)** Schematic of the DSP workflow for whole-transcriptome profiling: (1) transcriptome-wide UV-cleavable RNA probes were hybridized to mRNA; (2) samples were stained for GFP and PanCK, and probes were collected from GFP^+^, PanCK^+^, or GFP^−^/PanCK^−^ (Other) regions, yielding 153 areas of interest (AOIs); (3) collected probes were sequenced by next-generation sequencing (NGS). **(B)** Correlative intravital microscopy of a tongue containing four lesions after 24 weeks of 4NQO treatment. Insets show magnified images of clustering and non-clustering myeloid behaviors determined by IVM (left) and corresponding DSP sections (right) with enlarged views of the same regions. **(C)** Volcano plot showing differential gene expression between myeloid AOIs from clustered versus non-clustered lesions. A total of 5,972 myeloid-specific transcripts were analyzed (Fig. S5). Blue dots represent genes contributing to the enriched gene sets shown in (D). Dashed lines indicate significance thresholds (|log_2_FC| > 1, *p* < 0.05 by linear mixed model). **(D)** Gene Set Enrichment Analysis (GSEA) using the *Dictionary of Immune Responses* showing enriched profiles for monocyte, macrophage, and dendritic cell programs (FDR *q* < 0.25).

The predominant GFP^+^ myeloid population in both clustered and scattered infiltrates consisted of mDCs and CD74^+^ macrophages; however, these myeloid organizations differed in their capacity to recruit T cells. To determine whether these behavioral states could be distinguished transcriptionally, we performed GeoMx Digital Spatial Profiling (DSP) (Dong et al. 2025) on endpoint samples collected at 24 weeks (Fig. 4A). Six sections at varying depths spanning the epithelium and lamina propria were analyzed, including two lesions with scattered infiltrates and two with dense myeloid clusters that were spatially matched to intravital recordings (Fig. 4B). Across 153 areas of interest (AOIs), we profiled 19,962 genes covering lesions, the interstitial matrix, and adjacent healthy epithelium. Because DSP can exhibit limited spatial resolution compared to single-cell RNA sequencing (Dong et al. 2025), each gene was classified according to its predominant expression within GFP^+^ (myeloid), PanCK^+^ (epithelial), or GFP^−^/PanCK^−^ (“other”) AOIs. This approach identified 5,972 myeloid-associated genes, 11,548 epithelial genes, and 2,442 “other” genes (Fig. S5A). As validation, tumor epithelium showed upregulation of *Krt17* and downregulation of *Aldh3a1* relative to healthy tissue, confirming the neoplastic state (Yao et al. 2020; Liu et al. 2024) (Fig. S5B). Myeloid AOIs were enriched for antigen-presentation genes such as *Cd74*, *Ctss*, and *Cst3* (Bandola-Simon et al. 2025) (Fig. S5C), consistent with our mIF findings that the main infiltrating myeloid populations were antigen-presenting DCs and Mø^CD74+^ macrophages (Fig. 3D). Comparing myeloid clusters and non-clustered infiltrates (Fig. 4C) revealed distinct transcriptional programs. Scattered myeloid cells from progressing lesions expressed high levels of *Mmp12*, *ApoE*, and *LipA*, signatures associated with inflammatory remodeling, lipid metabolism, and M2-like polarization (Nelson et al. 2012). In contrast, dense myeloid clusters from regressing lesions were dominated by interferon-driven programs, including *Cxcl9*, *Cxcl10*, and interferon-stimulated genes such as *Isg15* and *Irf7*, known to mediate T-cell recruitment through CXCR3 in a positive feedback loop with T cell–derived IFNγ (Prizant et al. 2021).

Gene Set Enrichment Analysis (GSEA) using the *Dictionary of Immune Responses* (Cui et al. 2024) confirmed distinct functional polarization between myeloid states (Fig. 4D). Scattered myeloid cells from progressing lesions were enriched for Interleukin 1- and tumor necrosis factor α-responsive gene signatures, whereas clustered myeloid cells from regressing lesions were enriched for interferon-driven, T cell–recruiting programs. These profiles correspond to pro- and anti-tumorigenic environments, respectively, underscoring that intratumoral myeloid organization alone delineates the prevailing immune context. Interestingly, PD-L1 expression was higher in dense DC clusters than in scattered ones, suggesting a regulatory mechanism that modulates the timing and extent of antigen presentation to CD4 T cells. This also indicates that PD1/PD-L1–mediated immune suppression is not an initiating abnormality in the developing microenvironment of progressing lesions.

Together, these findings show that niche-forming DCs establish chemokine- and interferon-rich immune hubs that recruit and activate T cells, driving lesion regression. In contrast, scattered antigen-presenting cells adopt lipid-metabolic, akin to an M2-like program, that fail to recruit T cells and fosters immune evasion and lesion progression.

### Myeloid clusters predict lesion onset

**Figure 5 -:**
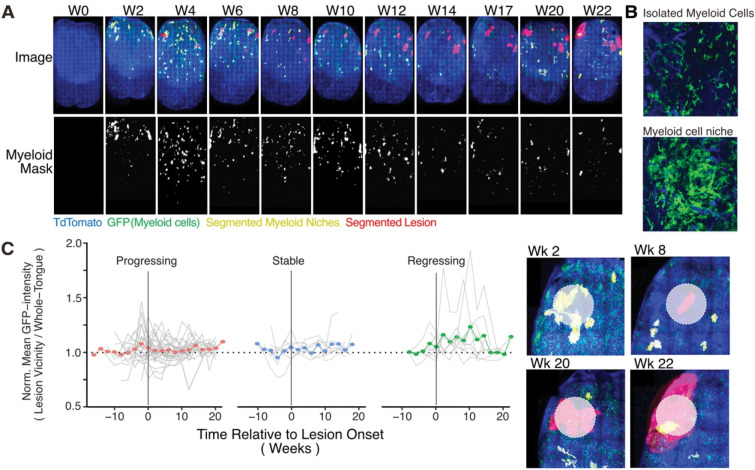
Myeloid clusters predict lesion onset. **(A)** Segmentation of myeloid clusters from sum-projected tongue images during the 4NQO treatment shows an early accumulation phase up to week 4, followed by a plateau between weeks 4 and 14, and a gradual decline thereafter. **(B)** Enlarged images comparing clustered myeloid cells with isolated individual cells. **(C)** Line graph showing GFP intensity within a 250 μm radius centered on each lesion, normalized to the average GFP level across the tongue. Gray lines represent individual lesions; colored lines and dots indicate the mean GFP intensity relative to adjacent healthy tissue. Distinct GFP elevation appears around progressing and stable lesions approximately two weeks before onset, but not for stable, lesions. The vertical line marks the first time point at which each lesion became morphologically detectable. Insets show examples of 250 μm radius regions overlaid on representative lesions.

We next asked whether myeloid cell behavior could predict lesion fate before visible lesions emerged. At the macroscopic level, we observed clusters of myeloid cells that were not associated with lesions at any treatment stage but were completely absent in healthy tongues (Fig. 5A–B). These transient myeloid niches accumulated during the first four weeks of 4NQO treatment (Fig. S6A), consistent with a response to early epithelial transformation rather than the acute DNA damage response that triggered neutrophil recruitment within two days (Fig. S4A). These early niches were composed primarily of DCs and CD74^+^ macrophages (Fig. S6B–D), often accompanied by CD4^+^ T cells, and mirrored the cellular composition of lesion-associated clusters. Quantification of the area occupied by these clusters across the ventral tongue showed a rapid increase during the first four weeks, followed by a plateau between weeks 4 and 14 and a decline thereafter, even before 4NQO withdrawal at week 16. This reduction likely reflects a systemic loss of myeloid clustering capacity rather than a direct effect of carcinogen cessation. Spatial transcriptomic analysis of these orphan niches at week 24 revealed upregulation of *Cxcl9*, *Cxcl10*, and *Cd74*, consistent with a T cell–recruiting phenotype (Fig. S6E).

Strikingly, lesion onset correlated with peaks in myeloid cluster activity. Spatial mapping showed that clusters were frequently localized to sites that later developed lesions (Fig. 5C). These findings suggest that such clusters represent early, abortive immune responses that fail to clear premalignant epithelial cells and thus mark sites predisposed to initial progression. Histological analysis further revealed that early clusters expressed high levels of PD-L1 (Fig. S6F) and recruited FOXP3^+^ regulatory T cells (Fig. S6G), establishing them as localized, immunosuppressive niches.

In summary, early myeloid clusters arise before lesion formation and selectively precede progressing and stable—but not regressing—lesions. Their transient, PD-L1–expressing, and Treg-enriched nature likely creates a permissive microenvironment for tumor initiation when premalignant epithelial cells escape immune clearance.

## Discussion

This study demonstrates that the spatial organization of myeloid cells alone predicts the fate of premalignant lesions. By longitudinally following individual lesions in an immunocompetent 4NQO carcinogenesis model, we identified three trajectories—progression, stability, and regression—each defined by distinct myeloid states. Dense DC clusters characterized regressing lesions, sparse, scattered antigen-presenting cells (APCs) marked progression, and basally confined or multifocal niche-forming APCs corresponded to stable lesions. Thus, lesion fate depends not on the presence of immune infiltrates but on their spatial and temporal organization.

Mechanistically, myeloid-derived DCs and CD74^+^ macrophages emerged as key regulators of early tumorigenesis. Confluent DC–macrophage niches expressed CXCL9 and CXCL10, were associated with T-cell recruitment, and formed interferon-responsive microenvironments linked to lesion clearance. Conversely, scattered DCs and CD74^+^ macrophages upregulated *Mmp12*, *ApoE*, and lipid-metabolic programs consistent with an M2-like polarization, failing to attract T cells. These results position myeloid-derived DCs as functionally plastic cells that toggle between immune activation and suppression during the earliest stages of neoplasia.

We also uncovered a close association between tissue metabolism and immune architecture. A decline in NADH fluorescence preceded myeloid infiltration in progressing lesions but followed it in regressing ones, indicating that metabolic shifts accompany, and may reflect, the evolving immune landscape. Neutrophil recruitment showed a similar pattern, concentrating peritumorally around low-NADH lesions but rarely entering the epithelium. Although the directionality of this relationship cannot yet be established, the NADH signal provides a robust landmark of lesion state, marking the transition between effective immune engagement and metabolic adaptation during premalignant evolution.

A key finding was the presence of transient PD-L1^+^/CXCL9^+^/CXCL10^+^ myeloid niches that appeared weeks before visible lesion formation. These aggregates—rich in APCs and FOXP3^+^ regulatory T cells—marked sites that more often progressed, yet they were not exclusive to them: approximately 20% of regressing lesions showed a similar transient pattern. While this trend did not reach statistical significance, it suggests that early myeloid clustering reflects a transient immune state that can resolve toward either clearance or tolerance, depending on local context. We interpret these early clusters as dynamic, spatially confined immune niches that may represent abortive or partially effective attempts at lesion control. This finding extends previous observations of DC–T-cell hubs in established tumors (Pelka et al., 2021; Magen et al., 2023; Oh et al., 2025) by showing that such structures can emerge before morphological transformation and mark sites at higher risk of progression. Their dual features of immune activation and suppression highlight a fragile equilibrium between rejection and tolerance at the threshold of malignant conversion.

These results have important implications for head and neck squamous cell carcinoma (HNSCC), a disease with over 800,000 new cases and ~450,000 deaths annually. Oral leukoplakia and dysplasia are recognized precursors, yet no reliable markers predict which lesions will regress or progress. The presence of transient PD-L1^+^/CXCL9^+^/CXCL10^+^ myeloid clusters at sites that later develop tumors suggests that immune organization itself may serve as an early biomarker—and potentially a therapeutic target—for cancer interception.

Our findings also reconcile conflicting views that myeloid niches can either support antitumor immunity or promote tumor growth. We propose that early interferon-driven, PD-L1^+^/Treg-rich myeloid clusters represent fragile immune microenvironments balanced between effective clearance and immune evasion. As lesions mature, dense T cell–recruiting clusters favor regression, whereas divergent myeloid differentiation produces sparse, lipid-reprogrammed DCs that drive progression. This framework integrates spatial organization, immune regulation, and metabolic state into a coherent model of premalignant fate.

Translationally, our findings open three avenues. First, imaging or molecular detection of CXCL9/10^+^ and PD-L1^+^ myeloid clusters could identify high-risk fields before invasive transformation. Second, therapies that promote myeloid niche formation or sustain interferon-driven programs could reinforce spontaneous regression and synergize with immune-checkpoint blockade. Third, advances in endoscopic multiphoton microscopy and metabolic imaging may soon enable longitudinal monitoring of immune architecture and NADH dynamics in patients, providing real-time tools for early detection and intervention.

In summary, spatiotemporal myeloid dynamics—rather than mutational burden alone—govern the outcome of premalignant lesions. Dense, immune-active DC niches promote regression; sparse, immunosuppressive DCs permit progression; and transient PD-L1^+^/CXCL9^+^/CXCL10^+^ clusters mark early, plastic immune states that can either resolve or evolve toward malignancy. These findings provide a mechanistic framework for spontaneous regression and a foundation for predicting and intercepting cancer at its inception.

## Materials and Methods

### Animals and procedures

All experiments were approved by the National Cancer Institute (National Institutes of Health, Bethesda, MD, USA) Animal Care and Use Committee and were compliant with all relevant ethical regulations regarding animal research*.* LysMcre (B6.129P2-Lyz2tm1(cre)Ifo/J) and mT/mG (B6.129(Cg)-Gt(ROSA)26Sortm4(ACTB-tdTomato,-EGFP)Luo/J were purchased from Jackson Laboratory and backcrossed at least 6 generations into c57Bl6/NJ background before crossed to homozygosity for both gene elements. All mice used in this study were between 8 and 14 weeks of age at the start of treatment. Mice were anesthetized by isoflurane inhalation (3%) followed by an initial intraperitoneal injection of a mixture of ketamine (100 mg per kg) and xylazine (20 mg per kg) in lactated Ringer’s solution before imaging sessions and maintained as necessary during imaging.

### 4NQO chemical induction of tumors

Premalignant lesions and tumors were induced as previously described(Amornphimoltham et al. 2017). In brief, 4-Nitroquinoline 1-oxide was administered at a concentration of 50μg/mL in the drinking water for 16 weeks (week 1–16) followed by a recovery period with regular water for 8 weeks (week 17–24). To maintain weight, the mouse diet was supplemented with a soft transgenic dough-diet (Bio-serv, S3472) once a week starting at week 5 of treatment. An equal number of male and female mice were used.

### Intravital Imaging

Two-photon microscopy was performed using an inverted laser-scanning two-photon microscope (MPE-RS; Olympus, Center Valley, PA) equipped with a tunable laser (Insight DSþ; Spectra Physics, Santa Clara, CA). The tongue of the anesthetized animal was gently placed in a 3D-printed tongue holder device (Wang et al. unpublished) with the ventral side facing the objective. The entire ventral area was imaged using an Olympus UPlanSApo IR 30x silicone oil objective (NA: 1.05, Evident) or a UPLXAPO 10x air objective (NA: 0.4, Evident) using a resonance scanner set to 3 line repetitions. The most superficial 150μm was acquired in each stack at a 2μm distance between optical slices (z-resolution). Excitation was performed at 900 and 740 nm, and the emitted light was collected by an appropriate set of mirrors and filters on three detectors (bandpass filters: blue for collagen or NADH: Z 410 to 460 nm; green for GFP: Z 495 to 540 nm; and red for TdTomato: Z 575 to 645 nm). The resulting stacks were stitched using the Fluoview software (Evident) after each imaging session. The same imaging setup was used for time-lapse imaging, except a 1.5x digital zoom and 6 line repetitions were used for an imaging depth of 72μm (49 optical sections at a z-resolution of 1.5μm per optical slice). 5–10 min of timelapse was acquired at a framerate of 19.4 3D stacks per second. High-resolution images were obtained in Galvano scanning mode using a UPLANSAPO 40X silicon immersion objective (NA: 1.40, Evident).

### Intravital Analysis

Stitched whole tongue 3D images were analyzed for lesion size using the Imaris v9.2.1 software (Oxford Instruments). First, the spatial locations of lesions were identified by three criteria: 1) altered epithelial basal cell morphology, 2) degree of collagen displacement compared to surrounding epithelium, and 3) maintained dysregulated morphology and/or displaced collagen for at least 3 timepoints in the same spatial location. To segment lesion volumes, the epithelial compartment was masked using the collagen channel as a reference, followed by manual outlining of the lesion. The collagen channel was first segmented using the surfaces tool setting smoothing to 10μm and local threshold to 2, selecting the largest surface from the results. The TdTomato channel was masked by the negative collagen surface, producing a channel with isolated epithelium. Each lesion was manually segmented in a 2-step process. First, the outline of the transformed epithelium in the masked TdTomato channel was manually annotated and masked using the surfaces tool; to generate a volumetric mask that follows the contour of the surface of the epithelium, the resulting lesion channel underwent an additional automated surface detection without smoothing and thresholds were set to 120 for S3 and S4 tongues, and 90 for S1 and SW tongues. The volume of the resulting surface was exported and analyzed using R. Lesion volumes were log2 transformed and fit to a linear equation over time in weeks using the lm() function (y=bx+a) and lesions with $>0.5\;r^{2}$ and b>0.1 were characterized as progressing. lesions were characterized as regressing when endpoint volume was ≤ 25% of the peak volume, and stable in cases where the fit was $<0.5\;r^{2}$ and b<0.1 but endpoint size was more than 25% of the peak volume.

To measure Myeloid displacement for each lesion, the GFP channel was masked using the established lesion segments using Imaris. Myeloid cells were segmented using the surfaces tool with smoothing set to 3 and local threshold set to 8, followed by a voxel filter of >=10. The resulting myeloid segment parameters within each were exported, and displacement was calculated as the total myeloid segment volume divided by the whole lesion volume. Myeloid behavior was determined through qualitative assessment as described in Figure 2.

Myeloid recruitment was measured using imageJ (NIH). Each tongue was imported using bioformats at resolution level 2. The 3D volumes were converted into sum-projects, and the papillae-less area was manually annotated. At timepoints prior to lesion onset, a circular region of interest with 250μm radius was drawn with the center in the spatial position of the lesion and GFP intensity was measured.

### Cardiac fixation and histological sectioning of the tongue

For cardiac perfusion, the left ventricle of the heart was punctured, and PBS supplemented with heparin (10U/mL, Hospira NDC0409–2720-30) was perfused to wash out the blood from the right atrium, followed by 10 ml fixative (4% paraformaldehyde in 200mM HEPES, pH 7.3). After a total of 10 min of fixation, the animal was further perfused with 10mL quenching buffer (20mM glycine, 50mM Ammonium chloride in PBS), 10mL sucrose (15% in PBS), and finally 10 mL sucrose (30% in PBS). The tongues were excised and incubated in 30% sucrose on ice until the tissue sank in the solution. The tongue was snap-frozen in a modified embedding medium for frozen sectioning (Dannhorn et al. 2020) (7.5% HPMC (40–60cP, sigma, H8384), 2.5% PVP(m/w Mn 360kDa, sigma PVP360) in water) on powdered dry-ice between two histology slides to maintain a flat orientation of the ventral epithelium for sectioning. The glass slides were removed by gentle heating with a thumb on the ventral side, and 5μm cryosections were cut (Leica CM1860 cryostat) and collected on charged microscopy slides (Globe, #1358).

### Multiplex Immunofluorescence staining and imaging

Slides retrieved from −80C were equilibrated first at −20C for 10min followed by 10 min at RT. Sections were post-fixed in 4% PFA (200mM HEPES pH 7.3) for 10 min at RT and washed 3 × 5 min (2x in 20 mM Glycine, 50mM NH_4_Cl in PBS and 1 x in PBS). Blocking was performed in 3% BSA, 5μg/mL Fc-Block (anti-CD15/32 TruStain FcX, Biolegend) for 1h at RT. For multiplex staining, the blocked sections were subjected to 6 rounds of the following procedure using different antibodies in each round (Round 1: CD45-AF75, CD8-AF647, Round 2: CD4-AF750, CD11c-AF647, Round 3: CD68-AF750, Ly6G-AF647, Round 4: LYVE1-Dy755, CD74-AF647, rabbit-anti Krt17-non conjugated. Round 5–1: Anti-rabbit-AF647, PanCK-AF555, ITGA6-Bio. Round5–2: Strep-AF750, MHC-II-AF594. Round 6: CD163-PE). For each round, slides were incubated with the antibodies diluted in 1% BSA, 1.25μg/mL Fc-Block for 2 h, followed by 3 × wash steps in PBS. Nuclei were stained using 5μg/mL Hoechst 33342 (Thermo Fischer Scientific) in PBS for 10 min followed by 2x wash steps in PBS and 2x wash steps in deionized water. Slides were mounted in Slowfade Diamond media (S36963, Thermo Fischer Scientific) and imaged using a Zeiss Axioscan Z.1 Slide Scanner equipped with a 20 × objective (Plan-Apochromat, NA 0.8) and a Colibri 7 Flexible Light Source. After imaging, the slides were retrieved, and the coverslip was removed by submerging in PBS for 2 h. Fluorophores were bleached using 1mg/mL LiBH4 in deionized water for 15 min, followed by 3 wash steps in PBS before initiating the following round of staining and imaging. During round 5, an additional bleaching step for TdTomato was included by incubating in 3.5% H2O2 in 90% MeOH on ice for 20 min after the LiBH_4_-bleaching and washing steps. Additionally, the antibody incubation step (Round 5–1) was followed by 3 × wash steps in PBS and an additional secondary antibody incubation step (Round 5–2) for 2h at RT.

Hematoxylin and eosin stainings were performed by VitroVivo.

### Image analysis of multiplexed images

#### Image Preprocessing –

Zeiss ZenBlue software was used to split each slide scan into individual tissues, and the czi files were loaded into the HALO v4.1 software (Indica Labs) for sequential stained image fusing and cell segmentation. Each round of tissue staining was merged by using the “register images” function using the Hoechst staining of the 6 sequential images for registration, followed by “fuse serial stain” with the Hoechst channel as reference and the first round as the image registration target. The resulting fused images were used for all downstream analysis. The outline of the ventral epithelial area and each lesion identified by IVM were manually annotated, and lesion annotations were subtracted from the intravitally imaged area to generate a “Healthy adjacent tissue” annotation.

#### Single Cell Segmentation –

A classifier pipeline containing four individual dense-net v2 AI-based classifiers was used to segment in-focus and correctly merged extracellular matrix and epithelial tissue areas. The first classifier to determine tissue from glass was trained on 5 annotations of tissue and glass from separate tissues using the TdTomato channels of the first round, and segments containing tissue were selected. The second classifier, designed to recognize in-focus vs out-of-focus tissue, was trained on 12 areas in focus and 28 out-of-focus areas from 12 tissues, and the in-focus segments were selected. The third classifier for detecting correctly merged vs incorrectly merged areas was trained on 12 correctly merged areas and 17 incorrectly merged areas from 3 sections for the training using the Hoechst channels from the 6 images, and the correctly merged segments were selected. The fourth classifier for classifying tissues into ECM, Epithelium, and Muscle segments was trained on 9 ECM areas, 13 muscle areas and 109 epithelium areas from 9 tissues, and both ECM and epithelium segments were selected the final analysis. To segment nuclei within each segment, a Nuclei Seg (Plugin) - FL v1.0.0 classifier was trained on 446 nuclei from 5 areas sampled from 5 different tissues. To extract single-cell fluorescence intensities of 24 markers (including 6 rounds of Hoechst channels and 4 rounds of TdTomato channels) a HighPlex FL v4.3.2 module was used on all annotations. This module implemented the trained classifier pipeline to identify tissue regions and the nuclear segmentation plugin to identify cells using a fixed maximal 2 μm cytoplasmic radius for each cell. Whole-cell intensities were exported and used for downstream analysis.

#### Single cell analysis –

Exported single cell values underwent unsupervised clustering for cell identity determination using phenograph as part of the “analysis of SPAtial single-Cell datasets” (SPAC) environment within the NIH Integrated Data Analysis Platform (Liu et al. 2025). First, batch correction was performed by applying arcsinh and z-score normalization. The normalized data was clustered using the phenograph node and transformed into the UMAP node in parallel. The resulting clusters were evaluated based on expression level, and all clusters with high expression of GFP, CD11c, CD68 and MHCII were pooled and underwent a second phenograph clustering to separate macrophages, dendritic cells, and misclassified epithelial cells. The percentage of immune cells within tumors was calculated using the epithelial segment of the manual annotations, and the results from different sections of the same lesion were summed before calculating the cell-specific prevalence.

### GeoMx Digital Spatial Profiling

Six sections from the same tongue of a female mouse that underwent the complete 24 weeks 4NQO-treatment regimen were collected with 25μm spacing between each section. For the NanoString GeoMx DSP RNA assays, slides were prepared following the Leica Biosystems BOND RX FFPE RNA Slide Preparation Protocol described in the GeoMx NGS Slide Preparation User Manual (NanoString, MAN-10 115–04). Briefly, slides were baked overnight at 60°C and then loaded into the Leica BOND RX device. Slides were treated sequentially following Leica BOND RX default HIER (ER2, 20 min, 95°C) Protocol and GeoMx DSP RNA Slide Prep Protocol (1 mg/mL proteinase K (Ambion, cat. 2546) in 1X phosphate-buffered saline (PBS) at 37°C for 15 min). After pretreatment, the slides were hybridized with the GeoMx Mouse Whole Transcriptome Atlas Mouse RNA for Illumina Systems (Nanostring, GMX-RNA-NGSMsWTA-4). The slides were dried of excess 1X PBS, set in a hybridization chamber lined with Kimwipes wetted with Diethyl pyrocarbonate(DEPC)-treated water, and covered with 200 μL prepared Probe Hybridization solution. HybriSlips (Grace Biolabs, cat. 714022) was gently applied to the slide, and incubated at 37°C overnight.

After hybridization, the HybriSlips were removed by dipping the slides in 2X saline-sodium citrate (SSC) (Sigma-Aldrich, cat. S6639)/0.1% Tween-20. To remove unbound probes, the slides were washed twice in Stringent Wash (50% formamide (ThermoFisher, cat. AM9342)/2X SSC) at 37°C for 25 min, followed by two washes in 2X SSC for 2 min. Slides were blocked in 200 μL Buffer W (NanoString), placed in a humidity chamber and incubated at room temperature for 30 min. The slide was dried of excess Buffer W, set in a humidity chamber, covered with morphology marker solution containing anti-GFP antibody (Abcam, Ab6673) and left to incubate at room temperature for 1 hour staining followed by 2x wash steps with SSC for 5 min. Secondary anti-goat antibody-594 and anti-pan-cytokeratin Alexa 532 antibody for 1 hour staining followed by 2x wash steps with SSC for 5 min. The sections were immediately loaded into the GeoMx instrument after staining.

### Digital Spatial Profiling analysis.

Briefly, RNA probe-hybridized and antibody-stained slides were scanned with a 20X objective, collecting data using FITC/525 nm (excitation 480/28 nm, emission 516/23 nm), Cy3/568 nm (excitation 538/19 nm, emission 564/15 nm), Texas Red/615 nm (excitation 588/19 nm, emission 623/30 nm), and Cy5/666 nm (excitation 645/19 nm, emission 683/30 nm) channels. Regions of interest (ROI) were selected from the corresponding intravital images based on morphology as well as cell surface marker staining. Within each large ROI, areas of interest (AOI) were identified for RNA collection for either Myeloid cells (GFP+), Epithelial cells (PanCK+), or “other” cells (PanCK−, GFP−). ROIs were annotated directly in the DSP software, and UV light was used to cleave the barcode linkers of the prebound RNA probes in each AOI, and the cleaved probes in DSP were retrieved into collection plates.

DSP collection plates were frozen and stored at −80°C. Plates were thawed at room temperature and libraries were prepared per manufacturer’s guidelines. Collection plates were sealed with a semi-permeable membrane and dried down at 65°C for 1 hour. Each well was resuspended in 10 μL DEPC-treated water, incubated at room temperature for 10 min, and then spun down. The library preparation was carried out in a 96-well PCR plates by mixing 2 μL PCR mix (NanoString), 4 μL of index primer mix (NanoString), and 4 μL of DSP sample. The following PCR program was used to amplify the Illumina sequencing compatible libraries: 37°C for 30 min, 50°C for 10 min, 95°C for 3 min, followed by 18 cycles of (95°C for 15 s, 65°C for 1 min, 68°C for 30 s), 68°C for 5 min and a final hold at 4°C. A total of 6 plates of 96 wells each were used.

The indexed libraries were pooled with an 8-channel pipette by combining 2 μL per well from the 12 columns (one 96-well plate) into 8-well strip tubes and then pooled into a 1.5 mL tube. The combined 50 μL pools were incubated with 60 μL SPRIselect beads (Beckman Coulter, cat. B23318) (1.2X bead to sample ratio) for 5 min in a 1.5 mL tube followed by standing on a magnetic stand for 5 min before removal of the supernatant. The beads were then washed twice with 200 μL of 80% ethanol and air dried for 3 min before being eluted with 50 μL elution buffer (10 mM Tris-HCl pH 8, 0.05% Tween-20, Teknova cat. T1485). Then a second round of SPRIselect beads (Beckman Coulter, cat. B2331860 ul; 1.2X bead to sample ratio) selection was carried out directly on the bead suspension as above. The washed beads were eluted in 20 μL elution buffer (10 mM Tris-HCl pH 8, 0.05% Tween-20, Teknova cat. T1485) and 18 μL of the supernatant was extracted to a new tube. The 18 μL of clean-up supernatant from each 96-well plate was pooled, and the library fragment size was assessed with the D1000 Tape Station assay (Agilent Technologies) and the expected size of ~162 bp was observed.

Total target counts per DSP collection plate for sequencing were calculated based on the NanoString DSP Worksheet. The target sequencing depth was 30 counts/μm^2^ for a total of 1.5B reads. Libraries were sequenced with a 100-cycle S1 kit on the NovaSeq 6000 at the CCR Sequencing Facility (Frederick, Maryland, USA).

## Supplementary Material

Supplement 1
